# The Relationship between Pain Catastrophizing and Cognitive Function in Chronic Musculoskeletal Pain: A Scoping Review

**DOI:** 10.1155/2023/5851450

**Published:** 2023-09-09

**Authors:** Cory Alcon, Elizabeth Bergman, John Humphrey, Rupal M. Patel, Sharon Wang-Price

**Affiliations:** ^1^High Point University, Department of Physical Therapy, High Point, NC, USA; ^2^Texas Woman's University, School of Physical Therapy, Dallas, TX, USA; ^3^Texas Woman's University, School of Physical Therapy, Houston, TX, USA

## Abstract

**Objective:**

Chronic musculoskeletal pain (CMP) poses a considerable threat to physical, mental, and financial health worldwide. Beyond physical difficulties, CMP has a pronounced impact on pain behaviors and cognitive function. The purpose of this scoping review was to examine the relationship between pain catastrophizing (PC) and cognitive function in CMP, identify gaps in the literature, and provide future directions for research on the topic.

**Methods:**

Search strings were entered in the following databases: PubMed, CINAHL, Nursing and Allied Health, Ovid Emcare, PsycInfo, and Scopus. Data from the included articles were extracted thematically based on diagnostic classification and included author(s), year of publication, country, aim, sample, methods, intervention (if applicable), and key findings.

**Results:**

30 articles were included after screening. The studied populations included patients with fibromyalgia, chronic low back pain, and CMP. Two studies were designed to assess the relationship between PC and cognition as the primary aim. The included studies demonstrated variable evidence regarding the relationship between PC and cognition. Only four studies included clinically relevant PC populations (i.e., Pain Catastrophizing Scale score >30), and all found significant correlations.

**Conclusion:**

Although evidence exists for the relationship between cognitive function and PC, there is a lack of rigorous research to indicate the strength of this relationship and the specific cognitive functions affected. The literature lacks appropriate populations needed to investigate clinically relevant PC and is limited by heterogeneous neuropsychological test batteries. Future research should include populations demonstrating the behaviors being studied, intentional analysis of outcomes, and appropriate cognitive tests.

## 1. Introduction

Chronic musculoskeletal pain (CMP) poses a considerable threat to the physical, mental, and financial health of populations worldwide with approximately one-third of the world's population living with some form of ongoing pain [1]. CMP encompasses a wide range of clinical diagnoses including, but not limited to, chronic low back pain (CLBP), whiplash-associated disorder, fibromyalgia, and widespread pain syndromes. As pain becomes chronic, healthcare utilization costs rise, wages are lost, and suffering progresses [2]. Of note, an overwhelming number of CMP diagnoses lack definitive structural disorders or pathology, making this the most poorly managed musculoskeletal condition [3–5]. As such, CMP appears to have a pronounced impact on many other domains including the cognitive-evaluative and motivation-affective components of pain. However, considering pain as a multidimensional process, it is difficult to assess and determine which specific characteristics contribute the most to the patient's presentation. In CMP, the cognitive-evaluative and emotional-affective processes such as pain catastrophizing and fear may impact the relationship between pain and cognitive function more than pain intensity alone [6].

The development of maladaptive pain behaviors, including pain catastrophizing, significantly impacts the trajectory of an individual's painful condition in the areas of overall health, quality of life, and functional outcomes. Pain catastrophizing increases the likelihood of greater pain severity, intensity, and disability caused by the painful condition, has been found to account for nearly half of the variance in the likelihood of developing chronic pain in people with CLBP, and is a strong predictor of future pain levels in people with CMP [7]. Specifically, pain catastrophizing is the most common maladaptive pain behavior seen in patients with chronic pain, as approximately 39% of patients with chronic pain report severe pain catastrophizing [8]. Pain catastrophizing is defined as “the tendency to magnify the threat value of pain stimulus and to feel helpless in the context of pain, and by a relative inability to inhibit pain-related thoughts in anticipation of, during, or following a painful encounter” [9]. Pain catastrophizing is associated with an increase in protective vigilance towards an individual's pain and is known to limit the success of common therapeutic interventions targeting peripheral tissues [10, 11]. As efforts have been made to target interventions for pain behaviors such as catastrophizing in people with CMP, these intervention effects on pain, quality of life, and function remain modest at best [12–14]. Targeted cognitive-based interventions have been developed to address these behaviors including choices such as pain neuroscience education (PNE), cognitive-behavioral therapy (CBT), and acceptance and commitment therapy (ACT). While the interventions do appear to have their greatest impact by reducing behaviors such as pain catastrophizing and fear, their effect sizes remain limited. Effects sizes even appear to significantly decrease when the level of specific behaviors reaches certain thresholds rendering the interventions less useful for the same targeted behavior [15, 16]. This may in part be due to the cognitively demanding nature of pain itself as evidence demonstrates that cognitive reserves are diminished by an upregulation of focus on current or anticipated pain [17–19].

Cognitive impairments such as deficits in attention, memory, learning, and decision making are present in nearly one-third of patients with chronic pain [20]. A 2018 systematic review by Higgens et al. showed a significant relationship between the occurrence of chronic pain and the presence of neurocognitive dysfunction, with significant, negative correlations between pain and performance on objective neurophysiological tests that assessed memory, attention, processing speed, and executive function (*r* = −0.301 to −0.698) [6, 21–23]. Higgens et al.'s findings support the idea that pain is multidimensional. The relationship between pain and cognitive function may be influenced by the affective and evaluative components of pain, such as pain catastrophizing, kinesiophobia, depression, and anxiety. Pain catastrophizing is likely to interfere with the efficacy of cognitive-based interventions as cognitive impairments may limit an individual's ability to amend the deleterious effects of catastrophic thoughts and behaviors [24, 25]. It has been hypothesized that catastrophizing leads to increased attentiveness towards pain or pain-related information [26–29]. Therefore, the cognitive resources directed towards an individual's pain may leave limited reserves for other cognitively demanding tasks such as participating in cognitive-based interventions.

Although two meta-analyses [30, 31] investigating attentional biases towards pain did not find specific relationships between catastrophizing and cognition, these analyses suggest that cognitive deficits may be unique to patients with specific pain profiles, likely including higher levels of pain catastrophizing. Therefore, the lack of effectiveness of cognition-based interventions may be due to the heterogeneity of pain populations. To date, only a few studies have explicitly explored the relationship between pain catastrophizing and cognitive function so as to better understand the potentially dynamic interplay between the two and provide insight into therapeutic targets. Evidence is growing to support the need to determine specific pain phenotypes that allows for improved assessment and management of CMP. More specifically, a deeper understanding of psychological phenotypes may help determine the best approach for and mechanisms of cognitive-based interventions [32–34]. Therefore, the primary aim of this scoping review was to examine the relationship between pain catastrophizing and cognitive function in CMP. The secondary aim was to identify the limitations of the literature and to provide future directions for research on the topic.

## 2. Methods

A scoping review was conducted to systematically identify and synthesize all available literature on the relationship between pain catastrophizing and cognitive function in people living with chronic pain [35]. In addition to summarizing literature, a scoping review can identify and map available emerging evidence and can report on the types of evidence in order to address and inform practice and future research [36]. Finally, being a scoping review, a thorough assessment of methodological quality or risk of bias was not performed [37]. To ensure the rigor of this scoping review, the procedures outlined in PRISMA-Extension for Scoping Reviews were followed [37]. The review was registered with the Open Science Framework (OSF) registry (osf.io/p2cqf).

### 2.1. Operational Definitions

For the purpose of this scoping review, we adopted the following operational definitions: (1) pain catastrophizing is a cognitive or emotional process encompassing magnification of pain-related stimuli, feelings of helplessness, and a generally pessimistic orientation [38], (2) cognition is the process by which the brain acquires, processes, stores, and retrieves information [39], and (3) cognitive function is the performance of the mental process of perception, learning, memory, understanding, awareness, reasoning, judgment, intuition, and language [40]. In addition, cognitive function was not defined in the context of pain.

### 2.2. Eligibility Criteria

The following criteria were determined *a priori* and were used to screen articles for this scoping review: (1) articles in English language, (2) studies that included adults with chronic musculoskeletal pain (defined as >3 months duration), (3) articles that were published from 1995 to 2022, (4) articles of primary sources only, (5) studies that contained an objective measure of pain catastrophizing, and (6) studies that contained an objective measure of cognitive function based on the abovementioned operational definition. Exclusion criteria included (1) studies consisting of experimentally induced pain and (2) non-scholarly products (e.g., magazine articles and editorials).

### 2.3. Databases and Search Strategy

A health science librarian (JH) led the search for this scoping review. The combination of potential search terms and subsequent synonyms was first identified and agreed upon by the scoping review team. Once a search string was finalized, a repository search was conducted to check for any existing reviews on the topic. As no such preexisting reviews were located, this scoping review therefore proceeded. The final selections of keywords and the search string are shown in Table 1. Modified search strings were utilized on the following databases: PubMed, CINAHL, Nursing and Allied Health, Ovid Emcare, PsycInfo, and Scopus, to incorporate additional controlled vocabulary phrases when and where possible. Where the database allowed, limiters were applied, including English language only, scholarly/academic journals only, and a date range from January 1995 to May 31, 2022. This date range was selected as it correlates to the year that pain catastrophizing was first specifically and objectively measured and reported in the literature.

Articles returned were uploaded to the systematic review tool, Rayyan (Rayyan Systems, Inc., Cambridge, MA). Next, Rayyan's duplicate detection tool was used to identify possible duplicates which were then manually inspected and removed where appropriate. Following the PRISMA guidelines [37], two researchers (CA and EB) utilized the “blind” function in Rayyan to independently screen each record by title and abstract and then by full text for its eligibility using the predetermined inclusion/exclusion criteria. When there was a conflict between the two researchers, a third researcher (SWP) resolved the conflicts.

After article screens were concluded, the researchers (CA and EB) performed a historical citation search of the included articles references and utilized Google Scholar's “cited by” tool to perform a forward citation search for any additional pertinent articles. Any new pertinent articles were added to the final list of the included articles. The citations included in these articles were also examined. Finally, the articles included in the review were searched for retractions and none were found.

### 2.4. Data Extraction

Data from the included articles were extracted and thematically organized based on diagnostic classification. Data extracted from each article included author(s), year of publication, country, aim, sample (including gender and age distributions when available), methods, intervention (if applicable), and key findings following the recommendations of the Joanna Briggs Institute's Reviewers Manual (JBI's Reviewers Manual) [41].

### 2.5. Synthesis of Results

A critical appraisal of study quality was not performed as this scoping review aimed to examine the relationship between pain catastrophizing and cognitive function in CMP and was not intended to examine intervention effectiveness. After initial review of the included articles by diagnostic categories, a second review was performed to investigate differences in those studies containing populations with clinically relevant pain catastrophizing versus those that did not. Synthesis of the data required an investigation into the outcomes used to measure pain catastrophizing and their cutoff scores. Finally, the specific neuropsychological tests performed in each study were reviewed to determine the proposed cognitive domain assessed.

## 3. Results

**Figure 1:**
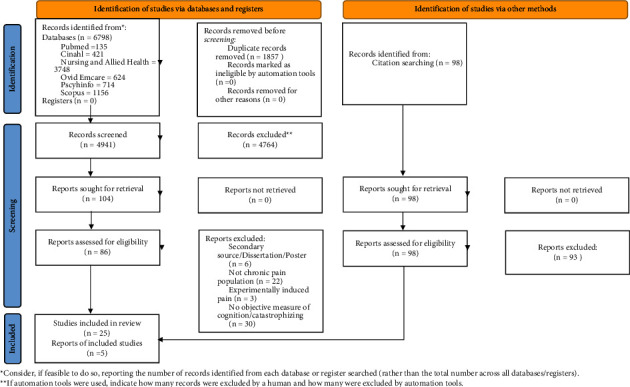
PRISMA flow diagram detailing database searches, abstracts screened, and full tests retrieved.

Figure 1 presents the PRISMA chart for the study selection, the screening process for each phase, and the reasons for study exclusion. After an initial search, 6798 articles were identified, and following the removal of the duplicates, 4941 articles were screened for eligibility. Consequently, 25 articles were included in this review after the initial screening. A follow-up citation search identified 98 articles, of which 5 were included for review. The 30 articles included in this review consisted of two randomized control trials (RCTs) and 28 observational studies. Studies were grouped according to the study diagnosis: 9 studies of fibromyalgia (FM), 10 studies of CLBP, and 11 studies of chronic musculoskeletal pain in other areas than low back. Study characteristics identified in the data extraction process are presented in Table 2 (FM), Table 3 (CLBP), and Table 4 (CMP).

The majority (*n* = 26) of the studies primarily used the Pain Catastrophizing Scale (PCS) [42] to assess the level of pain catastrophizing. However, four studies used the complete, subscale, or modification of the Coping Strategies Questionnaire (CSQ) [43], and one study used the Pain Response Self-Statements questionnaire (PRSS) [44]. A wide variety of neuropsychological tests were performed to measure cognitive deficits. The tests used to measure pain catastrophizing and the tests used to measure cognitive impairments and their specific intended targets (e.g., executive function, attention, and/or memory) are listed in Tables 2–4.

### 3.1. Fibromyalgia

FM is the most common diagnosis for which researchers investigate pain catastrophizing and impaired cognition [45]. For the FM population, one of nine studies by Galvez-Sánchez et al. [46] reported a significant relationship between catastrophizing and cognition (execution time, immediate recall, interference control, and recognition). In this study, the patients diagnosed with FM performed poorly on cognitive tests compared to healthy controls. Further, higher levels of catastrophizing explained the largest portion of the variance in poor cognitive performance (*r*^2^ *=* 0.39) [46]. Alternatively, a RCT tested a positive psychology Internet intervention versus a control condition and found that although the intervention reduced pain catastrophizing, it did not improve cognitive performance in executive tasks that involve attention, memory, and set shifting [47]. No associations were found between catastrophizing and task performance in those with FM in four studies [47–50]. However, the pain catastrophizing level was correlated with the occurrence of self-reported memory complaints in one study [51]. De Gier et al. [52] found that pain-related fear with concurrent high levels of pain catastrophizing was a strong predictor of cognitive performance, although there were no statistically strong correlations between catastrophizing and cognition. In addition, Moore et al. found a significant difference in attentional interference and catastrophizing between FM patients and healthy controls (*p* < 0.01), but correlational analyses were not performed to examine their relationship [53]. Similarly, Segura-Jiménez et al. reported gender differences in cognitive function and catastrophizing in this population without analysis of correlation [54].

### 3.2. Chronic Low Back Pain

CLBP contributes the most to disabilities related to musculoskeletal disorders and has a significant rate of recurrence [1]. In two studies, catastrophizing was found to be correlated with poorer cognitive performance (i.e., attention) on objective testing (*r* = 0.34, *p* < 0.05) [19, 55]. Similarly, attentional impairments demonstrated on reaction time tasks were also associated with pain catastrophizing in one study [56] and delayed trunk muscle activation during rapid arm movements in another study [57]. Melkumova et al. [58] also found pain catastrophizing to be associated with memory deficits assessed by a battery of cognitive tests in participants aged 51–60 years old (*r* = −0.495). In contrast, two studies found a significant difference in cognitive performance catastrophizing between LBP patients and controls but no significant correlation between these variables within the groups [59, 60]. Crombez et al. found that pain intensity, not catastrophizing, was predictive of attentional interference time [61]. One RCT by Baker et al. [62] showed that responders to a computerized cognitive training program had higher baseline pain catastrophizing. Lower self-reported cognitive function was correlated with a reduction in catastrophizing following the 8-week intervention. However, there was no correlation between objective cognitive testing and pain catastrophizing.

### 3.3. Other Chronic Musculoskeletal Pain

This group of studies (*n* = 11) was comprised of a population of syndromes or diagnoses that lack specific explanation as they relate to a definitive structure or tissue. Four studies found significant relationships between a high level of pain catastrophizing and poor cognitive function on a variety of tests [25, 63–65]. Three studies [66–68] found no association between pain catastrophizing and cognition, whereas four others identified the frequent presence of these variables together in CMP populations without testing specific relationships [69–72].

## 4. Discussion

Pain catastrophizing and cognitive dysfunction are common variables associated with CMP. These variables are known to negatively influence the management of the condition, but there is a limited understanding of how they interact to elicit that impact. The specific aim of this scoping review was to examine pain catastrophizing and its apparent connection with cognition. The results of this scoping review revealed no clear conclusions as to the relationship between pain catastrophizing and cognition. Only two studies [60, 70] specifically were designed to investigate this relationship. All other studies only included the relationship between the two variables of interest as secondary aims or included these two variables without primary consideration. Several themes arose during this scoping review including a lack of clinically meaningful pain catastrophizing levels within the study populations, limited utilization of pain catastrophizing subscale assessment, and variation in neuropsychological testing protocols.

Although several studies suggest that pain catastrophizing may impact cognitive performance in pain-free populations [73–76], the results of this scoping review were not conclusive. This may be in part due to the lack of definitions of clinically relevant levels of pain catastrophizing within the included studies. The accepted cutoffs for “clinically meaningful” pain catastrophizing are ≥30 points on the PCS [42, 77], ≥20 points on the CSQ [78], and >3.81 on the PRSS [44, 79]. Only four studies [46, 52, 57, 65] met the threshold for their respective pain catastrophizing measures, whereas 22 studies [47–51, 54, 56, 58–64, 66–72, 80] reported the mean scores less than the cutoffs in their participants, and four studies did not report the mean scores of their pain catastrophizing measures [25, 53, 81]. Without sample means for pain catastrophizing, results from these studies cannot be generalized to determine the relationship between clinically relevant pain catastrophizing and cognitive function. In addition, of the four studies reporting clinically meaningful levels of catastrophizing, three studies [46, 57, 65] reported negative correlations with a cognitive variable and one demonstrated a trend towards negative correlation [52]. Of the eleven studies [47–51, 59–61, 66–68] that reported their participants having a subclinical pain catastrophizing level, only five reported a significant relationship between pain catastrophizing and cognition [56, 58, 63, 64, 80], and six studies reported trends but no correlation analyses [54, 62, 69–72]. These findings may highlight an important gap in the literature as a majority of the populations studied lacked clinically relevant pain catastrophizing. Future research with larger sample sizes should investigate the relationship between pain catastrophizing and cognitive function within and between populations with high and low catastrophizing.

The most widely used outcome measure to capture the clinical presence of pain catastrophizing is the PCS [42]. The PCS provides four scores including a total score and the three subscale scores for rumination, magnification, and helplessness. Because the total PCS score can be impacted by its individual domains, it is important to consider the weight of those subdomains as well [9]. In addition to a cutoff score of 30 for the total score of the PCS, the cutoff scores of 11, 5, and 13 also have been determined for the rumination, magnification, and helplessness domains, respectively [42]. Any score equal to or higher than the cutoff scores indicates a significant clinical manifestation of pain catastrophizing overall or in the specific domain. Only three of the studies reviewed included subdomain scores in their analyses [25, 54, 65], and two studies found significant correlations between cognitive performance and PCS total scores, as well as the rumination subdomain [25, 65]. As the three domains of the PCS represent different constructs in the pain experience, the individual domain scores could further specify the areas of cognitive deficits [82]. The individual domain scores perhaps provide clinicians and patients more meaningful insights about their specific cognitive deficits to better plan an intervention strategy to address the deficits. Thus, the landscape of literature on this topic may paint an incomplete picture as to the relationship between cognitive function and pain catastrophizing when the relevance of individual subdomains is unknown.

One possible explanation for the inconclusive finding of the relationship between the pain catastrophizing level and cognition performance is due to a wide variety of neuropsychological tests used to assess cognitive function among the studies included in this review [20]. Although there are many validated tests for various cognitive functions, few are validated for populations with CMP and the reliability of a test can change when performed as part of a battery of other tests or can be subject to a varied amount of clinical judgment in its scoring [83]. Of the 30 articles reviewed, there were 45 different neuropsychological tests performed, none of which were consistently used across all studies.  Table 5 displays the tests used in the studies included in this scoping review and sorted by the cognitive function each test is proposed to assess. Attention and memory are the most commonly impaired cognitive functions in patients with chronic pain and were frequently tested in patients with pain catastrophizing [53, 84]. The high levels of variability in neuropsychological assessment among the studies in this review make it difficult to generalize the results. As research continues to expand in this area, researchers should state justification explicitly for the tests selected, and future studies should consider standardizing cognitive measures used for people living with CMP.

A majority (*n* *=* 26) of the studies included predominantly female samples with four of those recruiting only female participants. While the influence of gender was not a focus of this review, it is important to highlight that CMP does appear to influence females with greater frequency and severity than it does males [85]. Thus, the imbalance of gender within the included studies is not surprising. However, this does limit the ability to generalize findings across wider groups of people. Lastly, the mean age of study samples ranged from 21.8 to 70.3 years. Only one study included a sample >65 years of age [56] which is a typical cutoff used to control for the influence of age-related cognitive decline [86, 87].

## 5. Limitations

There are several limitations to this scoping review. Several of the included studies contained small sample sizes and many did not include pain-free comparison groups. These factors may limit the generalization of the findings as well as the ability to infer differences between CMP and pain-free populations. Although many of the articles included in this scoping review discussed descriptive relationships between pain catastrophizing and cognition, correlational analyses were either not performed or not mentioned. Therefore, there appear to be missed opportunities to demonstrate the relationship between the two variables of interest. The search strategy and inclusion/exclusion criteria used for this review attempted to limit the addition of studies investigating non-musculoskeletal pain diagnosis. In this process, there is the possibility that certain conditions, syndromes, or diagnoses were excluded based on the use of this term. Although the methods, searches, and review of this study were comprehensive and included blinding, there is potential for bias with retrieval and selection of research. Lastly, there is considerable variability in the definitions of cognition and its function, and it is therefore possible that certain domains of cognition function were not captured among the studies reviewed.

## 6. Conclusions

This scoping review shows that although evidence exists for the relationship between cognitive function and pain catastrophizing, there is a lack of rigorous research to indicate the strength of this relationship and the relationship of pain catastrophizing and specific cognitive deficits. Available literature contains small sample sizes of patients with clinically meaningful levels of pain catastrophizing, making it difficult to determine the utility of the results. There was also very little consistency in the neuropsychological tests used to investigate cognitive function. Future research should include a more comprehensive population that demonstrates the behaviors being studied, an intentional analysis of outcomes, and appropriate cognitive tests for chronic pain populations. Given the tremendous burden of CMP and its supported relationship with cognitive function, a better understanding of the deficits in specific cognitive functions will help promote improved clinical management and outcomes in the patient population with pain catastrophizing. Specifically, if cognitive-based interventions are standard practice in the management of pain behaviors such as pain catastrophizing and cognitive dysfunction is linked to those behaviors, treatment responses may be self-limiting. Thus, identifying psychological phenotypes in CMP patients and understanding appropriate intervention selection, sequencing, and progression based on those phenotypes may increase the likelihood of successful treatment outcomes in the CMP population.

## Figures and Tables

**Table 1:** Search string.

(“pain catastrophizing” OR “pain catastrophization” OR catastrophization OR catastrophizing OR catastrophize OR catastrophisation OR catastrophising OR catastrophise OR “pain vigilance” OR “pain rumination” OR “pain magnification” OR “pain helplessness”) AND (“chronic pain” OR “pain” OR “widespread pain syndrome” OR “fibromyalgia” OR “musculoskeletal pain”) AND (“cognitive function” OR “cognitive functioning” OR “cognitive skills” OR “cognitive ability” OR “cognitive abilities” OR “cognitive impairment” OR “executive function” OR “cognitive flexibility” OR “mental abilities” OR “mental ability” OR “cognitive dysfunction” OR “cognitively impaired” OR “executive dysfunction” OR “cognitive adaptability” OR “cognitive rigidity” OR “mental rigidity” OR “cognitive performance” OR “neuropsychological function” OR “cognition” OR “neurocognitive function” OR neurocognition OR “cognitive dissonance” OR “cognitive flexibility” OR “cognitive control” OR “executive control” OR “attention” OR “attentional control” OR “attentional interference” OR “attention deficit” OR “attention bias” OR “focus” OR “concentration”)

**Table 2:** Fibromyalgia articles.

Author(s), country, year	Aim(s)	Sample	Age and gender distribution	Methods	Intervention/manipulation	Measure of catastrophizing	Measure of cognition	Results	Clinically relevant catastrophizing (Y/N)
Boselie et al., Netherlands, 2018	Study the effect of a positive psychology intervention on negative states/traits and executive task performance	Patients with FM, *n* = 87–121	8 males, 234 females Mean age: 44.56	RCT	Positive psychology Internet intervention versus waiting list control condition	PCS	SST, DSTR, TST, WCST	Positive psychology intervention reduced pain catastrophizing but did not improve executive task performance	N

Castel et al., Spain, 2021	To compare cognitive performance and perception of memory problems and determine the relationship between memory complaints and cognitive performance	Patients with FM (*n* = 70), patients with chronic pain (*n* = 74), healthy controls (*n* = 40)	47 males, 137 females Mean age: 46.97	Observational	Groups based on diagnosis	Subscale of catastrophizing from CSQ	MFE, TAVEC, TPT, SCWT	Pain catastrophizing correlated with self-report memory complaints but not cognitive test performance	N

de Grier et al., Netherlands, 2003	To examine the role of pain-related fear and attentional processes on tolerance for physical activity	Patients with FM, *n* = 81	81 females Mean age: 48	Observational	Groups split into low and high fear groups based on Pain Anxiety Symptom Scale. Participants completed a physical performance task, cognitive task, and dual task	PCS	Card sorting and dual tasking	Pain-related fear was a strong predictor of cognitive performance, high levels of pain catastrophizing found in the high fear groups	Y

Dick et al., Canada, 2002	To investigate deficits in attentional functioning in pain groups	Patients with FM, chronic MSK pain, RA, and health controls, *n* = 20 per group	27 males, 53 females Mean age: 55.8	Observational	N/A	PCS	TEA	All pain groups had impaired cognition compared to controls but no correlation was found between catastrophizing and cognitive performance	N

Galvez-Sanchez et al., Austria, 2018	To investigate the contribution of affect-related variables to cognitive impairments	Patients with FM (*n* = 67) and health controls (*n* = 32)	99 females Mean age: 51.84	Observational	N/A	Subscale of catastrophizing from CSQ	ROCF, TAVEC, zoo map task, R-SAT, TMT	FM patients showed poorer cognitive performance than controls. Cognitive performance was associated with higher levels of catastrophizing, which explained the largest portion of variance in cognitive performance	Y

Miro et al., Spain, 2012	To explore the relationship between sex and cognitive alterations in those with FM	Patients with FM	20 males, 58 females Mean age: 46.61	Observational	N/A	PCS	ANT-I	No correlation between catastrophizing and cognition	N

Moore et al., United Kingdom, 2019	To examine attentional performance of FM patients compared to controls	Patients with FM (*n* = 24) and healthy controls (*n* = 26)	5 males, 45 females Mean age: 39.54	Observational	Participants performed cognitive tasks alone and during a painful pressure induction protocol	PCS	n-Back, attentional switching, and divided attention task	Participants with FM reported greater pain interference and catastrophizing compared to healthy controls. No correlations performed between cognition and catastrophizing	Unknown

Segura-Jimenez et al., Spain, 2016	To test gender differences in variables associated with FM and between those with or without FM	Patients with FM (*n* = 405) and without FM (*n* = 247)	74 males, 599 females Mean age: 48.5	Observational	N/A	PCS	MMSE, PASAT, and RAVLT	Inconsistent evidence of gender differences in FM-related symptoms. No correlation analyses performed	N

Veldhuijzen et al., Netherlands, 2012	To examine performance on cognitive tests in females with FM compared to controls	Female patients with FM (*n* = 35) and female healthy controls (*n* = 35)	70 females Mean age: 29.85	Observational	N/A	PCS	SCWT, MSIT, MMSE	Participants with FM had slower reaction times than controls but no difference in interference. No correlation between cognition and catastrophizing	N

**Table 3:** Chronic low back pain articles.

Author(s), country, year	Aim	Sample	Age and gender distribution	Methods	Intervention/manipulation	Measure of catastrophizing	Measure of cognition	Results	Clinically relevant catastrophizing (Y/N)
Baker et. Al., Australia, 2018	To investigate the relationship between objective and self-report cognitive impairments and the contribution of psychological attributes in participants with chronic back pain	Participants with chronic back pain, *n* = 41	17 males, 24 females Mean age: 42.97	Observational	N/A	PCS	TMT, SDMT, SCWT, WCST, TOPF, CFQ, EMQ, BRIEF-A	Catastrophizing correlated with poorer cognitive performance and most strongly related to objective cognitive performance	Unknown

Baker et al., Australia, 2018	To evaluate the potential efficacy of a computerized cognitive training to improve cognitive performance in participants with chronic pain	Participants with chronic pain, *n* = 39	15 males, 24 females Mean age: 43.3	RCT	Participants randomized to computerized cognitive training (*n* = 20) for 8 weeks (3x/wk) or active control intervention (*n* = 19)	PCS	TMT, SDMT, SCWT, WCST, n-back, CFQ, EMQ, BRIEF-A	Catastrophizing decreased in both groups, self-reported cognitive function was correlated with reduced catastrophizing, responders to the intervention had higher baseline catastrophizing	N

Crombez et al., Belgium, 2000	To investigate whether chronic pain patients display involuntary attentional shift towards pain-related information	Participants with chronic low back pain, *n* = 25	11 males, 14 females Mean age: 48.46	Observational	N/A	PCS	EST	Pain intensity, but not catastrophizing, was predictive of interference time	N

Goubert et al., Belgium, 2004	To investigate the effects of distraction from pain during and after a pain-inducing lifting task	Participants with chronic low back pain, *n* = 52	25 males, 27 females Mean age: 46.30	Observational	Participants performed a lifting task with and without a cognitively distracting condition. Order of tasks was randomized	PCS, researchers modified the CSQ to measure attention to pain and catastrophic thinking during the lifting task	Reaction time via random interval auditory tones	Catastrophizing was related to increased vigilance and less engagement in the distracting task. The effect of catastrophizing on cognitive performance was mediated by attention to pain	N

Jorge et al., Brazil, 2009	To investigate whether chronic low back pain and RA patients have deficits in memory	Participants with chronic low back pain (*n* = 21) and RA (*n* = 23)	8 males, 36 females Mean age: 55.03	Observational	N/A	CSQ	WMS III	Catastrophizing associated with memory deficits	Unknown

Lariviere et al., Canada, 2013	To examine the effect of pain and attentional interference on anticipatory postural adjustments in participants with chronic low back pain	Participants with chronic low back pain, *n* = 59	30 males, 29 females Mean age: 40	Observational	Participants performed rapid flexion movements of the right arm under 6 conditions including a control and different attentional demands. Assessed in groups by high/low catastrophizing	PCS	Latency of trunk muscle activation as a proxy of attentional interference	Catastrophizing and attention modulate trunk muscle activation	Y

Melkumova et al., Russia, 2011	To investigate the nature of impairments of cognitive functions and the factors influencing them in chronic pain	Participants with chronic spinal pain (*n* = 64) and healthy controls (*n* = 20)	22 male, 42 females Mean age: 49.1	Observational	Participants split by age: group 1—30–50 years and group 2—51–60 years	PCS	SNLCT, SDMT, SCWT, WCST, COWAT, DST, ROCF	In older participants (group 2), cognitive functions were most influenced by affective-motivation components of pain including catastrophizing	N

Roelofs et al., Netherlands, 2005	To examine the role of personal relevance of sensory pain-related words in selective attentional processing in chronic low back pain	Participants with chronic low back pain, *n* = 30	11 males, 19 females Mean age: 41.2	Observational	N/A	PCS	Modified SCWT	No correlation between cognition and catastrophizing	N

Simon et al., United States, 2016	To determine how working memory and catastrophizing are associated with pain intensity in chronic low back pain	Participants with chronic low back pain (*n* = 60) and pain-free controls (*n* = 30)	29 males, 61 females Mean age: 47.62	Observational	N/A	PCS	DSTR	Higher memory impairment and catastrophizing in chronic low back pain compared to controls but no correlation between the two	N

Tabira et al., Japan, 2020	To investigate the relationship between attention bias and psychological assessment	Participants with chronic low back pain, *n* = 13	2 males, 11 females Mean age: 70.3	Observational	Participants performed a reaction time task during an attentional bias modification task including neutral and threatening faces	PCS	Reaction time during ABM task	Catastrophizing associated with an increased attentional bias towards a threatening stimulus	N

**Table 4:** Chronic musculoskeletal pain articles.

Author(s), country, year	Aim	Sample	Age and gender distribution	Methods	Intervention/manipulation	Measure of catastrophizing	Measure of cognition	Results	Clinically relevant catastrophizing (Y/N)
BlaisdaleJones et al., Australia, 2021	To compare attentional biases, interpretation biases, and attentional control in people with and without chronic pain	Participants with chronic MSK pain (*n* = 74) and without chronic pain (*n* = 65)	66 males, 73 females Mean age = 49.53	Observational	Participants completed a visual scanning task with eye tracking, a recognition task, and a flanker task	PCS	VST, face task, word task, recognition task, flanker task	No correlation between catastrophizing and cognition	N

Coppieters et al., Australia, 2017	To examine grey matter alterations in patients with chronic whiplash compared to non-traumatic neck pain	Participants with chronic WAD (*n* = 31), chronic idiopathic neck pain (*n* = 34), and healthy controls (*n* = 28)	93 females Mean age: 33.52	Observational	N/A	PCS	PDQ, TMT	Greater self-report cognitive deficits in pain groups. Longer TMT times in pain groups but no difference in interference times. Higher catastrophizing in pain groups. WAD associated with decreased higher catastrophizing, poor cognitive function, and decreased GMV	N

Dehghani et al., Australia, 2004	To examine the ability of a cognitive-behavioral program to reduce attentional bias towards pain words	Participants with chronic MSK pain, *n* = 42	20 males, 22 females Mean age: 42	Observational	Participants completed a 3-week (5x/wk) intensive, multidisciplinary, day-patient program with 1 booster session 4 weeks following	PRSS	DPT	Fear of movement predicted attentional interference. No correlation found between catastrophizing and cognition	N

Dick et al., Canada, 2007	To examine how attention and memory are disrupted by chronic pain	Participants with chronic MSK pain receiving analgesic procedures, *n* = 24	6 males, 18 females Mean age: 47.55	Observational	Participants performed cognitive testing on days with and without an analgesic procedure	PCS	TEA, RST, SSP	Two-thirds of participants had disruption in attention and memory. No correlations performed between catastrophizing and cognitive variables	N

Giusti et al., Italy, 2020	To evaluate the role of psychological and executive function variables on chronic pain after orthopedic surgery	Participants undergoing orthopedic surgeries, *n* = 167	47 males, 120 females Mean age: 55	Observational	N/A	PCS	TMT	Catastrophizing and attention predict pain intensity. Catastrophizing predicts affective and evaluative components of pain	N

Gould et al., United States, 2021	To provide a detailed profile of veteran and community patients with chronic pain eligible for an implantable pain device	Participants within VA and community health systems with chronic MSK pain, *n* = 157	101 males, 56 females Mean age: 60.4	Observational	N/A	PCS	SLUMS	Pain catastrophizing and cognitive impairments are common in the populations	N

Grisart et al., Belgium, 2001	To explore memory performances of chronic pain patients	Participants with MSK pain, *n* = 18	10 males, 8 females Mean age: 43	Observational	N/A	PCS	CRT	Correlation between catastrophizing and controlled processing	Y

Lee et al., Korea, 2018	To examine how attentional engagement differs depending on psychological factors	Participants with chronic MSK pain, *n* = 50	18 males, 32 females Mean age: 21.8	Observational	Participants' attention to pain and anger-related stimuli were tracked using an eye tracker	PCS	Attentional focus via eye tracking	Increased catastrophizing correlated with increased focused time on pain stimuli	N

Legarreta et al., United States, 2016	To examine neuropsychological performance as it relates to specific aspects of pain	Participants with chronic MSK pain, *n* = 38	26 males, 12 females Mean age: 37.71	Observational	N/A	PCS	TMT, COWAT, SCWT, Ruff 2-7, CVLT	Negative correlation between catastrophizing and cognitive performance	Unknown

Mazidi et al., Iran, 2019	To investigate the relationship between and catastrophizing and attentional bias	Participants with chronic MSK pain (*n* = 28) and pain-free controls (*n* = 29)	18 males, 39 females Mean age: 34.07	Observational	Participants observed pictures of pain, happy, and neutral facial expressions while gaze behavior was recorded	PCS	Attention via eye tracking dwell time on painful, happy, and neutral faces, ATTC	Higher catastrophizing and low attentional control lead to decreased dwell time on happy faces	N

Oostermann et al., Netherlands, 2012	To examine different aspects of executive and attentional control in chronic pain with the confounding role of psychomotor control	Participants with chronic MSK pain (*n* = 34) and healthy controls (*n* = 32)	19 males, 47 females Mean age: 53.45	Observational	N/A	PCS	SCWT, TMT, BVT, ZMT	Impaired attentional performance in chronic pain but no correlation between cognition and PC	N

PCS = Pain Catastrophizing Scale, CSQ = Coping Strategies Questionnaire, PRSS = Pain Response Self-Statements, SST = stop signal task, DSTR = digit span task reverse, TST = task switching test, WCST = Wisconsin card sorting test, MFE = memory failures in everyday questionnaire, TAVEC = Test de Aprendizaje Verbal Espana-Complutens, TPT = Toulouse-Pieron perceptual and attention test, SCWT = Stroop color and word test, TEA = test of everyday attention, ROCF = Rey–Osterrieth complex figure test, ZMT = zoo map test, R-SAT = revised strategy application test, TMT = trail making test, ANT-I = attentional network test-interactions, MMSE = mini mental state exam, PASAT = paced auditory serial addition test, RAVLT = Rey auditory verbal learning test, MSIT = multisource interference test, SDMT = symbol digit modalities test, TOPF = test of premorbid function, CFQ = cognitive failures questionnaire, EMQ = everyday memory questionnaire, BRIEF-A = brief rating inventory of executive function-adult, EST = emotional Stroop task, WMS III = Wechsler memory scale III, SNLCT = sequential number-letter combination test, COWAT = controlled oral word association test, DST = digit span test, ABM = attentional bias modification, VST = visual scanning task, PDQ = perceived deficits questionnaire, DPT = dot probe test, RST = reading span task, SSP = spatial span task, SLUMS = Saint Louis University Mental Status Examination, CRT = cued recall test, Ruff2-7 = Ruff 2 and 7 selective attention test, CVLT = California verbal learning test, ATTC = attention control scale, and BVT = Bourdon-Vos test.

**Table 5:** Domains of neuropsychological tests.

Attention	Memory	Other
Stop-signal task Toulouse-Pieron perceptual and attention test Stroop color word test Dual tasking Test of everyday attention Trail making test Attentional network test-interactions Attentional switching Divided attention task Multisource interference test Emotional Stroop test Number sequence repetition test Visual scanning task Face task Word task Recognition task Flanker task Dot probe task Ruff 2 and 7 Selective attention test Bourdon-Vos test	Reverse digit span Rey–Osterrieth complex figure test California verbal learning test n-Back test Rey auditory verbal learning test Test of premorbid function Wechsler memory scale III Digit span backward test Reading span test Spatial span test Cued recall task	*General* Mini mental state exam BRIEF-A SLUMs *Planning* Zoo map task Revised strategy application test Sequential number-letter combination test *Processing speed* Paced auditory serial addition test Symbol digit modalities test Auditory tones reaction time Latency of trunk muscle activation *Set shifting* Task shifting Wisconsin card sorting *Verbal fluency* Verbal associations test

## Data Availability

Data from this review are available from the corresponding author upon request.
